# Factors Associated with Intention to Be Vaccinated Against Seasonal Influenza in Healthcare Workers: A Cross-Sectional Study Assessing Health Belief Model Constructs in Serbia

**DOI:** 10.3390/vaccines14090763

**Published:** 2026-09-01

**Authors:** Larisa Vujnovic, Biljana Kilibarda, Sofija Jovanovic, Dragana Dimitrijevic, Milunka Milinkovic, Zoran Bukumiric, Verica Jovanovic

**Affiliations:** 1 Institute of Public Health of Serbia “Dr Milan Jovanovic Batut”, 11000 Belgrade, Serbia; 2 Mediterranean and Black Sea Programme in Intervention Epidemiology Training (MediPIET), European Centre for Disease Prevention and Control (ECDC), 17183 Stockholm, Sweden; 3 Radiology Centre, University Clinical Centre of Serbia, 11000 Belgrade, Serbia; 4 Independent Researcher, 11000 Belgrade, Serbia; 5 Faculty of Medicine, University of Belgrade, 11000 Belgrade, Serbia

**Keywords:** health behaviours, vaccine hesitancy, influenza vaccine, intention, healthcare workers

## Abstract

**Introduction:** As seasonal influenza vaccination coverage among healthcare workers (HCWs) in Serbia remains suboptimal, we aimed to identify HCW beliefs associated with their intention to receive seasonal influenza vaccine (“vaccination intention”) in the season 2024/2025. **Methods:** In a cross-sectional survey conducted in 2024, healthcare institutions were selected from a national registry, using multi-stage sampling stratified by geographical region and healthcare level. All consenting HCWs present on the survey day self-completed questionnaires. Five dichotomised Health Belief Model constructs were assessed: perceived susceptibility to influenza, perceived disease severity, perceived vaccination benefits, perceived physical and psychological barriers, and perceived cues for action (recommendation by another HCW, someone close, or a supervisor). The association of vaccination intention (yes/no/undecided) with Health Belief Model constructs, demographic and occupational factors were assessed using multinomial logistic regression. **Results:** Of 1919 respondents, 30% declared positive vaccination intention, 52.5% no intention, and 17.5% were undecided. Compared with no intention, positive vaccination intention was associated with perceived self-benefit (aOR = 6.22, CI = 3.49–11.08), perceived patient benefit (aOR = 2.26, CI = 1.48–3.45), perceived susceptibility (aOR = 3.26, CI = 2.19–4.86), and perceived vaccine safety (aOR = 2.95, CI = 1.93–4.50). Participants were more likely to be undecided than reject vaccination if they experienced higher perceived susceptibility (aOR = 2.93, CI = 1.89–4.54), perceived influenza as severe (aOR = 2.04, CI = 1.30–3.21), perceived patient benefits (aOR = 1.77, CI = 1.14–2.74), or recalled receiving a recommendation from another HCW (aOR = 2.63, CI = 1.17–5.85). **Conclusions:** HCWs’ positive vaccination intention was associated with beliefs relevant for personal health and patient benefits. The undecided group showed a distinct pattern of beliefs warranting their separate analysis and specific approaches.

## 1. Introduction

Seasonal influenza is a substantial global health burden; estimates indicate that globally, influenza-attributable lower respiratory tract infections result in around 9.5 million hospitalizations [1] and approximately 290,000–650,000 influenza-associated deaths annually [2]. In healthcare settings, seasonal influenza presents an additional burden for the health system, as well as for the patients [3,4]. In a review of 14 observational studies, 11.4% of influenza cases were hospital acquired and were more likely to experience severe outcomes than the non-hospital-acquired cases [5]. Similarly, an analysis of four influenza seasons in adults admitted to a tertiary hospital in Spain reported approximately two times higher proportions of severe clinical outcomes in nosocomial influenza cases, compared with the community acquired ones [6]. Nosocomial influenza can originate from various sources, including infected HCWs [4]. At the same time, occupational exposure may place HCWs at increased risk of influenza infection. There are examples of healthcare workers (HCWs) caring for infected patients who may experience infection rates exceeding 50% during the transmission season [4] or even during the periods without influenza epidemic activity [7]. Without distinguishing the setting where the infection was acquired, a 2011 review encompassing 97 influenza seasons reported that influenza infection rates among HCWs were as high as 19% among unvaccinated HCWs compared with approximately 6% among vaccinated HCWs during seasonal epidemics [8]. Among influenza prevention and control measures, vaccination of healthcare workers against seasonal influenza remains widely recommended, both to protect HCWs themselves and for its broader benefits [9].

Influenza vaccination coverage among HCWs remains suboptimal: a meta-analysis across 26 countries reported an overall uptake of approximately 42%, including similarly low rates within Europe [10]. In Serbia, vaccination rates among HCWs fluctuated between 13% and 31% from 2018 to 2023 [11,12,13,14,15,16]. Vaccination against seasonal influenza is mandatory for HCWs meeting specified risk and patient-contact criteria under the national immunisation programme; the regulation does not prescribe a sanction for non-compliance [17]. HCW seasonal influenza vaccination is covered by mandatory health insurance [18].

Individuals vary in terms of vaccine acceptance, from firmly accepting or firmly rejecting vaccination to being “undecided” [19,20]. Undecided individuals may differ from those with a clearer preference; factors associated with their decisions about vaccination may require different interventions to increase acceptance [21,22]. Studies have shown that the undecided individuals more readily shift towards accepting vaccination than those who firmly reject it, while firm rejecters may shift to being undecided more readily than directly shifting to firm acceptance [20,23]. Vaccine acceptance depends on complex, often interrelated factors; in addition to personal and professional characteristics and knowledge, attitudes and beliefs play a major role [24,25,26]. Various frameworks are available for studying these determinants [27,28,29]. A widely used framework for examining vaccine acceptance is the Health Belief Model (HBM), which encompasses a range of constructs such as perceived susceptibility, perceived severity, perceived benefits, perceived barriers and cues for action [30]. Several studies have used HBM to assess the factors associated with influenza vaccination behaviour or intention among HCWs [31,32]. To the best of our knowledge, this framework has not been applied to a similar outcome in HCWs in Serbia. Understanding factors associated with different vaccination intentions in this setting may support the development of targeted interventions to improve influenza vaccination uptake among HCWs.

Guided by the Health Belief Model, this study aimed to explore factors associated with the intention to receive seasonal influenza vaccination and with being undecided about vaccination, compared with having no intention to be vaccinated against seasonal influenza, among healthcare workers in Serbia.

## 2. Materials and Methods

We conducted a cross-sectional study among HCWs in Serbia who were employed at the healthcare institutions included in the “Plan of Healthcare Institutions Network of the Republic of Serbia” [33]. This network comprises all publicly owned healthcare institutions in Serbia. The sampling frame was derived from the national Registry of Health Care Institutions and Health Care Workforce, which is maintained by the Institute of Public Health of Serbia “Dr Milan Jovanovic Batut”. The selection of institutions as the primary sampling units was stratified by region and by healthcare level. Institutions were selected proportionally to the number of employees, with potential alternatives in case a selected institution was unable to participate during the data collection period. The sample included three healthcare levels (primary, secondary and tertiary) and four statistical regions of Serbia (Belgrade region, Vojvodina region, Sumadija and West Serbia region, and South and East Serbia region), as defined by the decree on statistical territorial units nomenclature [34]. These regions correspond to the NUTS-2 level (Nomenclature of territorial units for statistics) used by Eurostat. The initial sample size was calculated using a precision-based approach (Slovin’s formula), assuming a population size of 78,766 and a 5% margin of error; after adjusting for a design effect of 3.9, the obtained sample size was 1553.

Data were collected between 20 and 24 May 2024 using a questionnaire developed under a project within the Partnership for International Vaccine Initiatives (PIVI) to assess HCWs’ knowledge, attitudes, practices and beliefs related to seasonal influenza vaccination (Supplementary Material File S1). The questionnaire was administered in Serbian. Some questions were adapted from the WHO guide, Behavioural and social drivers of vaccination: tools and practical guidance for achieving high uptake, using the questions originally developed for COVID-19 vaccination [35]. The adapted questions were back-translated into English by two independent interpreters and the two versions were reconciled. No significant differences were identified between the back-translated and original versions. The questionnaire underwent a two-stage pretesting process. First, it was cognitively pretested among seven HCWs to assess the clarity, comprehensibility, relevance, and appropriateness of the questions and response options. The revised questionnaire was subsequently piloted among 45 HCWs to assess the feasibility of questionnaire administration and identify any remaining problems with its content, format and completion. Necessary modifications were made after each stage before final implementation. Eligible participants were HCWs employed at participating healthcare institutions who were present at work on the data collection day within the data-collection period. They were invited to participate anonymously by completing the paper-based questionnaire which was distributed by designated coordinators nominated by each healthcare institution and collected in survey boxes.

Measures of demographics, attitudes, and beliefs regarding vaccination, as well as medical history relevant for the study, were collected. Occupation was collected according to medical specialty and dichotomised for analysis into the following groups: physicians/dentists and nurses/medical technicians. Vaccination beliefs were collected and analysed through the HBM constructs: perceived susceptibility to influenza (being worried about the possibility to get influenza), perceived severity of influenza (agreeing that influenza can cause severe illness or death), perceived physical barriers to vaccination (perceived easiness of vaccination), perceived psychological barriers to vaccination (perceived safety), and perceived benefits of vaccination for oneself (agreeing influenza vaccination is important for one’s health) and for the patients (agreeing HCW vaccination can reduce influenza in their patients). Cues for action were also assessed through three variables: recommendation by a supervisor, recommendation by another HCW and recommendation by someone close. Constructs in the HBM were dichotomised into yes or no responses for perception variables. Each recommendation variable was dichotomised into having received the recommendation once or more, versus not having received it. The outcome was assessed by asking participants, “do you intend to get vaccinated against flu during the next season 2024/2025?” Responses were categorised into three groups; intending to get vaccinated (“yes”), intending to not get vaccinated (“no”), and being undecided (“I am not sure”). Summary of the variables is available in Supplementary Material Table S1.

NUTS—Nomenclature of territorial units for statistics; HBM—Health Belief Model; HCW—healthcare worker. *Information on workplace was collected by the research coordinator.

All statistical analyses were performed in SPSS version 22. Univariable and multivariable multinomial logistic regression were used to test the association of the HBM constructs with the intention to get vaccinated against seasonal influenza (yes, no, undecided), with “no intention to get vaccinated” as the reference outcome. Candidate variables for the logistic regression model were selected a priori based on the HBM theoretical framework, empirical evidence from similar studies, and relevance for a potential public health intervention. Nested models were then compared using the likelihood ratio test. Variables that did not significantly improve the model fit were excluded to obtain a more parsimonious model, provided that their removal did not impair the stability of the remaining coefficients. Both age and professional experience were considered as variables and were strongly correlated, indicating collinearity. Since age is positively associated with more severe influenza outcomes [36], it was considered as a potential confounder for HBM constructs such as perceived severity and perceived benefits for own health and was therefore retained in the final model. The final model included age, region, occupation, and HBM constructs. Statistical significance was set at *p* < 0.05 and the results are presented as crude and adjusted odds ratios (ORs) with 95% confidence intervals (95% CIs).

The study is part of a project within the Partnership for International Vaccine Initiatives (PIVI). The study design and data collection have been reviewed and approved by the Ethical Committee of the Institute of Public Health of Serbia “Dr Milan Jovanovic Batut”, Approval No. 1323/1 from 6 March 2024.

## 3. Results

A total of 1919 HCWs responded to the survey, among whom 1541 (81.6%) were female and 1509 (79.0%) were medical technicians or nurses. Characteristics of the participants are presented in Table 1. The mean age of the respondents was 43.3 ± 11.9 years. The intention to get vaccinated in the following season was reported by 572 (30%) respondents, while 1001 (52.5%) declared having no intention to get vaccinated, and 333 (17.5%) were undecided.

In the univariable logistic regression, the constructs associated with the intention to get vaccinated, as opposed to no intention, were as follows: lack of perceived physical barriers (vaccination being perceived as easy), low perceived psychological barriers (vaccination being perceived as safe), perceived susceptibility to influenza and perceived severity of the disease, perceived benefits both for one’s own health and the patients’ health, and the recollection of a single or repeated recommendation by another healthcare worker, a supervisor, or someone close (Table 2). Older age was associated with the intention to get vaccinated as was personal medical history of chronic illness or recent mild influenza (in the previous five years). Male participants were more likely to report that they intended to get vaccinated. Additionally, physicians and dentists had higher odds of having the intention to get vaccinated compared to nurses and medical technicians.

Factors associated with being undecided versus not intending to be vaccinated are shown in Table 3. Physicians were more likely to be undecided compared to not intending to be vaccinated than nurses and medical technicians. Perceived safety, perceived benefits (both for oneself and for the patients), perceived susceptibility, and perceived severity of the disease were associated with higher odds of being undecided toward vaccination compared to not intending to be vaccinated. Persons who recall having received one or multiple recommendations from another healthcare worker, or from someone close, as well as a single recommendation from a supervisor, had a higher odds of being undecided about vaccination. No association was found for logistic ease of vaccination (lack of physical barriers).

In the multivariable multinomial logistic regression analyses (Table 2 and Table 3), believing that the seasonal influenza vaccine is important for one’s own health was associated with more than six-fold higher odds of intending to get vaccinated compared with declaring no intention. Perceived vaccine safety was associated with approximately three times higher odds of vaccination intention versus no intention. Increasing age was also associated with higher odds of intending to receive the influenza vaccine, although the magnitude of the association was small.

HCWs who perceived that patients benefited when HCWs are vaccinated had higher odds of intending to get vaccinated or being undecided. Similarly, higher perceived personal susceptibility to influenza was associated with a higher odds of intending to get vaccinated or being undecided compared to declaring no intention to get vaccinated.

Perceived disease severity was only associated with increased odds of being undecided versus having no intention. Recommendation from another HCW was associated with being undecided versus not intending to be vaccinated.

## 4. Discussion

This study used the Health Belief Model as the theoretical framework to explore factors associated with the intention of healthcare workers in Serbia to get vaccinated against seasonal influenza. Although undecided individuals may represent a distinct group, they are often analysed jointly with those who reject vaccination. In the present study the undecided HCWs were analysed separately to examine the factors associated with being undecided compared to having no intention to get vaccinated against seasonal influenza.

Several beliefs relevant to personal health were associated with vaccination acceptance, defined as the intention to get vaccinated in the next influenza season: perceived benefit for one’s own health, perceived susceptibility of influenza, and perceived vaccine safety. Additionally, perceived benefit to the patients was independently associated with the intention to get vaccinated, as was receiving a recommendation from a close person. Perceived patient benefits and perceived susceptibility also differed between the undecided and those expressing no intention to get vaccinated, while there was no difference in perceived safety or perceived self-benefit between these two groups. Perceived disease severity and recommendation from another HCW were associated with being undecided rather than having no intention to be vaccinated. Our findings support treating undecided HCWs as a distinct group.

Perceived susceptibility to influenza and perceived benefit to patients were common to both HCWs who intended to be vaccinated and those who were undecided, distinguishing both groups from those who firmly did not intend to be vaccinated. Existing evidence supports our finding that HCWs who receive influenza vaccinations are more likely to perceive it as beneficial for the health of their patients [37,38,39,40,41]. Our finding that higher perception of the benefits to patients distinguishes the undecided from those who firmly do not intend to be vaccinated adds new insight to the existing evidence, as few studies have examined people who have not made a definite decision.

An association between high perceived susceptibility and higher influenza vaccination intention or uptake has been widely reported [37,38,39,41,42] and has also been confirmed in our study. Beyond quantitative associations, the literature on qualitative research offers additional insights into how these beliefs may be articulated. In a qualitative study among nurses, perceiving oneself as being in good health emerged as a reason for rejecting vaccination [43].

In contrast, the perceived benefit for one’s own health was associated with firm vaccination acceptance but not with being undecided. The association between perceived self-benefit and influenza vaccination acceptance is consistent with previous studies [38,44].

Similarly, higher perceived vaccine safety was associated with greater odds of vaccination acceptance compared with firm rejection. Several studies have reported a similar association for influenza vaccination among HCWs [38,39,44,45], although none of these studies examined undecided participants separately. Low perceived vaccine safety as a psychological barrier to vaccination is not limited to influenza. Reservations regarding vaccine safety among HCWs have been reported for various vaccines [46,47].

In contrast, perceived ease of vaccination was not associated with vaccination acceptance. This suggests that, in this sample, perceived psychological rather than physical barriers were associated with vaccination intention.

The small positive association between increasing age and vaccination acceptance is consistent with previous studies of influenza vaccination among HCWs [37,45] and is partly consistent with findings on the COVID-19 vaccination among HCWs [48].

Beyond the factors shared with HCWs who intended to get vaccinated, the undecided group showed a distinct pattern of associations. In our study, higher perceived disease severity was associated with being undecided. Published research shows a positive association of perceived disease severity with past seasonal influenza vaccination among HCWs in Singapore [45], while a Canadian study found a similar association with the uptake of the pandemic influenza vaccine [38]. Receiving a recommendation from another HCW was also associated with being undecided rather than having no intention to get vaccinated, but it was not associated with firm vaccination intention. A previous study in Toronto found a positive association between a recommendation from another HCW and previous influenza vaccination among physicians in training [42], while HCWs in an Algerian study identified colleagues as a source of information about influenza vaccines [49]. These findings need to be interpreted with caution, as cues for action may be susceptible to potential recall bias.

Although HCWs who are undecided about vaccination are often omitted from data collection or analysis, most commonly by collapsing the responses into strictly positive and negative categories [22], they have been included as a separate category in this study. Since intention and behaviour may change in stages [50] and the undecided individuals may be more influenced by the negative attitudes towards vaccination in certain contexts [51], this group warrants communication strategies specific to the factors associated with uncertainty.

### Limitations

A limitation of the study was that the number of HCWs invited to participate was not recorded, so the response rate could not be calculated and variations in response rate between strata could not be assessed. The geographical distribution of respondents was skewed compared to that of the overall HCW population employed at the institutions constituting the sampling frame. Furthermore, only the public healthcare institutions were included in the study and private institutions were not represented. The data collection period was in May, outside of the influenza season.

Notwithstanding these limitations, the study offers insight through a theory-driven approach, using the Health Belief Model to guide variable selection and interpretation. To our knowledge, it represents one of the first studies of influenza vaccination intention among HCWs in Serbia using this framework. The inclusion of HCWs who were undecided about vaccination as a distinct group enhances the relevance of the findings for the design of targeted public health interventions. The relatively large and diverse sample, drawn from multiple institutions, professions, and regions, supports the contextual relevance of the results.

## 5. Conclusions

This study identified several patterns of beliefs associated with influenza vaccination intentions among healthcare workers in Serbia and supports treating undecided HCWs as a distinct group rather than combining them with those who firmly reject vaccination. Perceived benefit to one’s own health and perceived vaccine safety were particularly associated with firm vaccination intention. Perceived susceptibility to influenza and perceived benefit to patients were associated with both vaccination acceptance and being undecided, rather than firm rejection, suggesting that these beliefs may be relevant for remaining open to vaccination. Perceived disease severity was specifically associated with being undecided, while different cues for action were associated with firm acceptance and indecision.

These findings suggest that influenza vaccination interventions for HCWs may benefit from recognising different patterns of beliefs according to vaccination intention, rather than treating all those who have not made a definitive decision to get vaccinated as a homogeneous group. Communication addressing vaccine safety and personal health benefits may be particularly relevant to firm acceptance, while approaches for undecided HCWs could additionally address perceptions of influenza risk and the benefits to patients. Recognising undecided HCWs as a distinct group may help inform more targeted and supportive vaccination communication.

## Figures and Tables

**Table 1:** Characteristics of the participants.

Characteristic			
		Mean	Standard deviation
Age (years)		43.3	11.9
Professional experience (years)		18.8	12.2
		Frequency	%
Gender	Male	348	18.4
	Female	1541	81.6
Region	Belgrade	293	15.3
	Vojvodina	496	25.8
	Sumadija and West Serbia	653	34.0
	South and East Serbia	477	24.9
Occupation	Physician or dentist	402	21.0
	Nurse or medical technician	1509	79.0
Having been diagnosed with influenza in the last 5 years	Yes, severe	94	4.9
	Yes, mild	776	40.8
	No	1030	54.2
Has a chronic illness	Yes	506	26.8
	No	1380	73.2
Intention to get vaccinated against influenza	Yes	572	30.0
	Undecided	333	17.5
	No	1001	52.5

Missing values were observed for age (n = 35), professional experience (n = 18), gender (n = 30), occupation (n = 8), having been diagnosed with influenza in the last 5 years (n = 19), presence of a chronic illness (n = 33), and intention to get vaccinated against influenza (n = 13).

**Table 2:** Factors associated with the intention to get vaccinated against seasonal influenza in the 2024/2025 season compared to having no intention to get vaccinated.

Associated Factor	Category	“Yes, I Intend to Get Vaccinated”, n (%)	“No, I Don’t Intend to Get Vaccinated”, n (%)	OR ^§^ (95% CI)	aOR ^§§^ (95% CI)
Age (years), mean ± SD	Per year	46.4 ± 11.2	42.0 ± 11.8	1.03 (1.02–1.04) ***	1.04 (1.02–1.06) ***
Professional experience (years), mean ± SD	Per year	21.5 ± 12.0	17.8 ± 12.1	1.001 (0.990–1.011)	—
Gender	Male	122 (42.5)	165 (57.5)	1.37 (1.06–1.78) *	—
	Female (ref.)	442 (35.0)	821 (65.0)	Ref.	—
Region	South and East Serbia	133 (35.8)	239 (64.2)	2.76 (1.87–4.06) ***	1.37 (0.74–2.53)
	Vojvodina	170 (43.5)	221 (56.5)	3.81 (2.60–5.58) ***	4.06 (2.14–7.70) ***
	Šumadija and West Serbia	225 (41.1)	323 (58.9)	3.45 (2.39–4.98) ***	1.96 (1.09–3.55) *
	Belgrade (ref.)	44 (16.8)	218 (83.2)	Ref.	—
Occupation	Physician or dentist	183 (55.0)	150 (45.0)	2.66 (2.08–3.41) ***	1.29 (0.83–1.99)
	Nurse or medical technician (ref.)	388 (31.4)	846 (68.6)	Ref.	Ref.
Having been diagnosed with influenza in the last 5 years	Mild influenza	254 (40.5)	373 (59.5)	1.34 (1.08–1.66) **	—
	Severe influenza	28 (36.4)	49 (63.6)	1.12 (0.69–1.82)	—
	No influenza (ref.)	289 (33.7)	568 (66.3)	Ref.	—
Having a chronic illness	Yes	180 (41.6)	253 (58.4)	1.36 (1.08–1.71) **	—
Perceived physical barrier	Vaccination perceived as easy: Yes	532 (37.9)	870 (62.1)	1.98 (1.33–2.95) **	0.78 (0.38–1.60)
Perceived psychological barrier	Vaccine perceived as safe: Yes	431 (61.7)	268 (38.3)	8.66 (6.79–11.03) ***	2.94 (1.92–4.48) ***
Perceived susceptibility to influenza	Worried about contracting influenza: Yes	317 (66.3)	161 (33.7)	6.54 (5.16–8.28) ***	3.27 (2.20–4.87) ***
Perceived severity of influenza	Influenza may cause severe illness or death: Yes	420 (49.7)	425 (50.3)	3.97 (3.13–5.03) ***	1.08 (0.69–1.69)
Perceived self-benefit	Vaccine important for one’s health: Yes	534 (54.3)	449 (45.7)	18.78 (12.99–27.16) ***	6.29 (3.53–11.18) ***
Perceived patient benefit	Vaccination reduces the possibility of patients contracting influenza: Yes	366 (63.1)	214 (36.9)	6.78 (5.36–8.56) ***	2.30 (1.51–3.50) ***
Supervisor recommendation	Once or more	227 (40.1)	339 (59.9)	1.60 (1.27–2.01) ***	1.26 (0.73–2.19)
	None (ref.)	222 (29.6)	529 (70.4)	Ref.	Ref.
HCW recommendation	Once or more	184 (50.4)	181 (49.6)	2.46 (1.90–3.19) ***	1.01 (0.54–1.88)
	None (ref.)	217 (29.2)	526 (70.8)	Ref.	Ref.
Close person recommendation	Once or more	103 (58.9)	72 (41.1)	3.28 (2.35–4.59) ***	2.32 (1.22–4.39) *
	None (ref.)	258 (30.4)	592 (69.6)	Ref.	Ref.

OR—odds ratio; aOR—adjusted odds ratio; CI—confidence interval; Ref.—referent group; HCW—healthcare worker. * *p* < 0.05; ** *p* < 0.01; *** *p* < 0.001. ^§^ 1573 participants were included in this analysis; participants answering “undecided” were excluded from this analysis; missing values within the analysed subset: gender (n = 23), occupation (n = 6), having been diagnosed with influenza in the last 5 years (n = 12), presence of a chronic illness (n = 18), vaccination perceived as easy (n = 27), perceived vaccine safety (n = 57), perceived susceptibility to influenza (n = 15), perceived severity of influenza (n = 96), perceived self-benefit (n = 19), perceived patient benefit (n = 80), supervisor recommendation (n = 256), HCW recommendation (n = 465), close person recommendation (n = 548). For dichotomised variables, the “no” category was used as the referent group. ^§§^ Missing values n = 883; the final multivariable model was selected as the most parsimonious model, based on model fit and stability of the regression coefficients; aORs are shown only for variables retained in the final multivariable model.

**Table 3:** Factors associated with being undecided about getting vaccinated against seasonal influenza in the 2024/2025 season compared to having no intention to get vaccinated.

Associated Factor	Category	Undecided, n (%)	“No, I Don’t Intend to Get Vaccinated”, n (%)	OR ^§^ (95% CI)	aOR ^§§^ (95% CI)
Age (years)	Per year	42.1 ± 12.1	42.0 ± 11.8	1.00 (0.99–1.01)	0.99 (0.98–1.01)
Professional experience (years)	Per year	17.2 ± 12.6	17.8 ± 12.1	1.000 (0.998–1.001)	—
Gender	Male	59 (26.3)	165 (73.7)	1.10 (0.79–1.52)	—
	Female (ref.)	268 (24.6)	821 (75.4)	Ref.	—
Region	South and East Serbia	104 (30.3)	239 (69.7)	3.39 (2.15–5.34) ***	1.87 (1.00–3.51)
	Vojvodina	99 (30.9)	221 (69.1)	3.49 (2.20–5.52) ***	3.12 (1.62–6.00) ***
	Šumadija and West Serbia	102 (24.0)	323 (76.0)	2.46 (1.56–3.86) ***	1.80 (0.97–3.35)
	Belgrade (ref.)	28 (11.4)	218 (88.6)	Ref.	Ref.
Occupation	Physician or dentist	67 (30.9)	150 (69.1)	1.43 (1.04–1.97) *	0.77 (0.47–1.25)
	Nurse or medical technician (ref.)	264 (23.8)	846 (76.2)	Ref.	Ref.
Having been diagnosed with influenza in the last 5 years	Mild influenza	146 (28.1)	373 (71.9)	1.33 (1.03–1.72) *	—
	Severe influenza	16 (24.6)	49 (75.4)	1.11 (0.62–2.00)	—
	No influenza (ref.)	167 (22.7)	568 (77.3)	Ref.	—
Having a chronic illness	Yes	71 (21.9)	253 (78.1)	0.83 (0.61–1.12)	—
Perceived physical barrier	Vaccination perceived as easy: Yes	306 (26.0)	870 (74.0)	1.55 (0.98–2.44)	0.77 (0.41–1.46)
Perceived psychological barrier	Vaccine perceived as safe: Yes	169 (38.7)	268 (61.3)	2.90 (2.23–3.76) ***	1.35 (0.88–2.08)
Perceived susceptibility to influenza	Worried about contracting influenza: Yes	138 (46.2)	161 (53.8)	3.70 (2.81–4.88) ***	2.94 (1.90–4.54) ***
Perceived severity of influenza	Influenza may cause severe illness or death: Yes	216 (33.7)	425 (66.3)	2.65 (2.02–3.48) ***	2.08 (1.33–3.26) **
Perceived self-benefit	Vaccine important for one’s health: Yes	261 (36.8)	449 (63.2)	4.52 (3.37–6.07) ***	1.56 (0.98–2.47)
Perceived patient benefit	Vaccination reduces the possibility of patients contracting influenza: Yes	153 (41.7)	214 (58.3)	3.20 (2.45–4.18) ***	1.72 (1.12–2.67) *
Supervisor recommendation	Once or more	125 (26.9)	339 (73.1)	1.34 (1.02–1.76) *	0.59 (0.33–1.06)
	None (ref.)	146 (21.6)	529 (78.4)	Ref.	Ref.
HCW recommendation	Once or more	111 (38.0)	181 (62.0)	2.52 (1.86–3.42) ***	2.23 (1.22–4.09) **
	None (ref.)	128 (19.6)	526 (80.4)	Ref.	Ref.
Close person recommendation	Once or more	49 (40.5)	72 (59.5)	2.47 (1.65–3.70) ***	1.37 (0.72–2.62)
	None (ref.)	163 (21.6)	592 (78.4)	Ref.	Ref.

OR—odds ratio; aOR—adjusted odds ratio; CI—confidence interval; Ref.—referent group; HCW—healthcare worker. * *p* < 0.05; ** *p* < 0.01; *** *p* < 0.001. ^§^ 1334 participants were included in the analysis; participants answering “yes” were excluded from this analysis. Missing values within the analysed subset: gender (n = 21), occupation (n = 7), having been diagnosed with influenza in the last 5 years (n = 15), presence of a chronic illness (n = 23), vaccination perceived as easy (n = 23), perceived vaccine safety (n = 58), perceived susceptibility to influenza (n = 13), perceived severity of influenza (n = 90), perceived self-benefit (n = 18), perceived patient benefit (n = 75), supervisor recommendation (n = 195), HCW recommendation (n = 388), close person recommendation (n = 458). For dichotomised variables, the “no” category was used as the referent group. ^§§^ Missing values (n = 883); the final multivariable model was selected as the most parsimonious model, based on model fit and stability of the regression coefficients; aORs are shown only for variables retained in the final multivariable model.

## Data Availability

Consistent with the data management and sharing provisions specified in the protocol for which ethical approval is obtained public access to the respondent-level dataset is not provided.

## Supplementary Materials

The following supporting information can be downloaded at https://www.mdpi.com/article/10.3390/vaccines14090763/s1, Supplementary File S1: English translation of the survey questionnaire; Table S1: Variable description.

## Abbreviations

The following abbreviations are used in this manuscript:

CI	confidence interval
ICU	intensive care unit
HCW	healthcare worker
HBM	Health Belief Model
OR	odds ratio
aOR	adjusted odds ratio
SD	standard deviation
