# Supplementary material for: Factors Associated with Intention to Be Vaccinated Against Seasonal Influenza in Healthcare Workers: A Cross-Sectional Study Assessing Health Belief Model Constructs in Serbia
Source: Vaccines (Basel). 2026 Sep 1;14(9):763. doi: 10.3390/vaccines14090763 (PMC13611065; doi:10.3390/vaccines14090763)
Supplement: Supplementary file 1 [file vaccines-14-00763-s001.zip › Supplemenatry material Table S1.pdf]

Supplementary Table S1. Variable description.

Variable	Description
**Demographic – personal**	
Age	Years (continuous)
Gender	Categorical, dichotomous<br>Male/Female
**Professional**	
Occupation	Categorical, dichotomised for analysis<br>Physician or dentist versus<br>Nurse or medical technician
Professional experience	Years (continuous)
Region (workplace)*	Categorical, four NUTS-2 level regions<br>Belgrade (capital city region)<br>Vojvodina<br>Sumadija and West Serbia<br>South and East Serbia
**Personal medical history**	
Has a chronic illness	Categorical, dichotomous<br>Yes/No
Has been diagnosed with influenza in the past five years	Categorical, three levels<br>No/Yes, mild influenza/Yes, severe influenza
**Vaccination beliefs (Health Belief Model constructs)**	
Perceived psychological barriers to vaccination (Do you agree with the statement "Influenza vaccine is safe"?)	Categorical, dichotomised for analysis
Perceived physical barriers to vaccination (How easy is it for you to get vaccinated against influenza from an organisational/practical aspect (physical distance of vaccination point, waiting time, etc.)?)	Categorical, dichotomised for analysis
Perceived self-benefit of vaccination (How important for your health is the influenza vaccine in your opinion?)	Categorical, dichotomised for analysis
Perceived benefit of vaccination for the patients (Do you agree with the statement "Vaccination of HCWs may reduce influenza infections in their patients"?)	Categorical, dichotomised for analysis
Perceived severity of influenza (Do you agree with the statement "Influenza can lead to severe disease or death")	Categorical, dichotomised for analysis
Perceived susceptibility to influenza (How worried are you about the possibility to get ill from influenza"?)	Categorical, dichotomised for analysis
Cue for action – recommendation by someone close to get vaccinated	Categorical, dichotomised for analysis<br>None/Once or more
Cue for action – recommendation by a supervisor to get vaccinated	Categorical, dichotomised for analysis<br>None/Once or more
Cue for action – recommendation by another HCW to get vaccinated	Categorical, dichotomised for analysis<br>None/Once or more

**Outcome**<br>Intention to get vaccinated<br>Do you intend to get vaccinated against flu during the next season 2024/2025?	Categorical, three levels<br>“Yes”, being undecided “I am not sure”, “No”.
