# Supplementary material for: Factors Associated with Intention to Be Vaccinated Against Seasonal Influenza in Healthcare Workers: A Cross-Sectional Study Assessing Health Belief Model Constructs in Serbia
Source: Vaccines (Basel). 2026 Sep 1;14(9):763. doi: 10.3390/vaccines14090763 (PMC13611065; doi:10.3390/vaccines14090763)
Supplement: Supplementary file 1 [file vaccines-14-00763-s001.zip › Supplementary material File S1.pdf]

INSTITUTE OF PUBLIC HEALTH OF SERBIA “DR MILAN JOVANOVIC BATUT”
English translation of survey questionnaire

QUESTIONNAIRE FOR THE STUDY OF KNOWLEDGE, ATTITUDE AND BEHAVIOUR OF HEALTH WORKERS IN RELATION TO VACCINATION AGAINST INFLUENZA

We kindly ask you to carefully read the questions and complete the questionnaire. The questionnaire does not collect any personal data. Participation is ANONYMOUS and VOLUNTARY.

Date of completing the questionnaire: _______________

Name of health institution: ____________________________________

Type of health institution (e.g. primary health centre, hospital, clinical centre etc.): ______________

Municipality: ____________________

I SOCIODEMOGRAPHIC AND DATA ON WORKING PLACE

1. Sex:
a. Male
b. Female

2. Age:
__________(please write a number)

3. What is your profession?
a. Medical doctor specialist (please specify specialization):
b. Medical doctor, in residency (please specify residency/specialization):
c. Medical doctor (not a specialist and not in residency/specialist training)
d. Doctor of stomatology (with or without specialisation)
e. Laboratory technician
f. Medical nurse/medical technician
g. Dental nurse/ technician
h. Radiology technician
i. Physiotherapist
j. Other (please specify)

4. In which department/organizational unit do you work? (If you are working at several departments please chose/specify the one where you spend most working hours)

a. General practice
b. Laboratory
c. Internal medicine
d. Intensive care unit
e. Emergency service
f. Gynaecology/obstetrics
g. Paediatric dep.
h. Infectious diseases dep.
i. Pulmonology
j. Haematology
k. Oncology
l. Surgery
m. Other:

5. On average, how many patients do you interact with per day?

____________________(please give the average number)

6. How many years of experience in health care do you have? (disregarding if the experience is from several health institutions)

____________________(number of years)

7. Do you have a chronic condition diagnosed by a medical doctor?

a. Yes
b. No

8. Do any of the following household members have a chronic disease/condition diagnosed by a medical doctor? If there is no such person in your household please chose the response: "There is no such person in my household".

		Has a chronic disease	Does not have a chronic disease	No such person in my household
8.1	Newborn/infant (0 – 1 years)	1	2	3
8.2	Pre-school child	1	2	3
8.3	School child	1	2	3
8.4.	Persons aged 18 to 64	1	2	3
8.4	Persons aged 65 or older	1	2	3

II PERSONAL EXPERIENCE WITH INFLUENZA AND VACCINATION

9. Have you had influenza in the past 5 years (including this season), regardless of whether it was laboratory-confirmed? (multiple answers are possible in case that you had influenza more than once in the last 5 years)

a. No
b. Yes, I had a mild form of the disease
c. Yes, I had pneumonia, but I was not hospitalized
d. Yes, I was hospitalized because of influenza

10. Have you ever been vaccinated against influenza?
a. Yes
b. No

11. How many times have you got influenza vaccination in the last 4 years (excluding this season)?
I have received influenza vaccine______________ times in the last four years

12. Have you been vaccinated against influenza in this season 2023/2024?
a. Yes
b. No

13. Do you intend to get vaccinated against influenza in the next season 2024/2025?
a. Yes
b. No
c. I am not sure

14. Have you ever been vaccinated against COVID-19?
a. Yes
b. No

15. Have you ever been vaccinated against hepatitis B?
a. Yes
b. No

III ACCESSIBILITY AND OTHER FACTORS IN RELATION TO INFLUENZA VACCINATION

16. Is there organized vaccination against influenza for staff in the health institution in which you work?
a. Yes, every season
b. Yes, sometimes
c. No

17. If there is no specially organized vaccination against influenza in the institution where you work, do you know where you could get vaccinated against influenza?
a. No
b. Yes_________________________________(please specify where)

18. How easy/convenient is it for you to get vaccinated against influenza? (distance from the vaccination site, long waiting times etc.)
a. Not easy at all
b. Somewhat easy
c. Moderately easy
d. Very easy

19. Has any of the following people recommended and/or requested that you get vaccinated against influenza?

		Yes, once	Yes, more than once	No
19.1	Your superior	1	2	3
19.2	A health professional	1	2	3
19.3	A close person (i.e. household member, partner...)	1	2	3

IV KNOWLEDGE AND PRACTICE on IFNLUENZA and INFLUENZA VACCINATION

20. “In your opinion, is influenza vaccination in our country mandatory, recommended, or neither for the following population groups? (Please circle the response that, in your opinion, is correct for each population group)

		Mandatory	Recommended	Neither mandatory nor recommended	I don’t know
20.1	Pregnant women	1	2	3	4
20.2	Persons older than 6 months with diagnosis of chronic disease	1	2	3	4
20.3	Immunocompromised persons	1	2	3	4
20.4	Elderly (65 years and older)	1	2	3	4
20.5	Employees in health care institutions	1	2	3	4

21. Can a person infected with influenza virus transmit the diseases to another person even if there are no symptoms/asymptomatic?
a. Yes
b. No
c. I don’t know

22. Time needed for acquiring immunity after influenza vaccination is...
a. A week
b. Two to three weeks
c. A month

23. Post-vaccinal immunity after influenza vaccination lasts …
a. 3 to 5 months
b. From 6 to 12 months
c. Two years
d. Life long

24. Have you in the last five years (including this year) established a clinical diagnosis of influenza in at least one patient, regardless if the diagnosis was laboratory confirmed?
a. Yes
b. No
c. Not applicable (this is not part of my job responsibilities)

25. Have you in the last five years (including this year) diagnosed influenza which was laboratory confirmed in at least one patient?

a. Yes
b. No
c. Not applicable (this is not part of my job responsibilities)

26. In the current season (2023/2024), have you personally recommended influenza vaccination to at least one person in the following population groups?

		Yes	No	Not applicable
26.1	Healthy adults	1	2	3
26.2	Pregnant women	1	2	3
26.3	Persons with chronic diseases	1	2	3
26.4	Immunocompromised persons	1	2	3
26.5	Elderly persons (65 years and older)	1	2	3
26.5	Employees in healthcare institutions	1	2	3

V BELIEFS AND ATTITUDES ON INFLUENZA VACCINATION

27. In your opinion, how much is influenza vaccination important for your own health?

a. Not important
b. Somewhat important
c. Moderately important
d. Very important

28. How concerned are you about the possibility to get influenza?

a. Not at all concerned
b. A little concerned
c. Moderately concerned
d. Very concerned

29. “Do you think that most of your family and friends want you to get vaccinated against influenza?

a. Yes
b. No

30. How much do you agree that the following factors are important for your decision to get vaccinated against influenza? (please circle one response in each row)

		Strongly disagree	Disagree	Neither agree nor disagree	Agree	Strongly agree
30.1	Vaccine producer (company)	1	2	3	4	5
30.2	Country of origin of the vaccine	1	2	3	4	5
30.3	Type of influenza vaccine (inactivated, trivalent, quadrivalent, live attenuated …)	1	2	3	4	5

31. If you had the option to choose which influenza vaccine to receive, which of the following would you choose?"

a. Vaccine produced in Serbia (for example "Torlak")
b. Vaccine from a foreign producer

32. How much do you agree with the following statements?
(circle one response in each row)

	Statement	Strongly disagree	Disagree	Neither agree nor disagree	Agree	Strongly agree
32.1	Vaccine against influenza is safe	1	2	3	4	5
32.2	Vaccine against influenza is efficacious, it can prevent getting the disease	1	2	3	4	5
32.3	Vaccination against influenza can protect from severe disease or complications	1	2	3	4	5
32.4	Vaccination against influenza can reduce risk from hospitalization due to influenza disease	1	2	3	4	5
32.5	Vaccination against influenza reduced death outcomes in case of influenza disease	1	2	3	4	5
32.6	Vaccination against influenza can cause a person to get influenza (become sick)	1	2	3	4	5

33. How much do you agree or disagree that the following institutions/persons may influence your decision to get vaccinated? (circle one response in each row)

		Strongly disagree	Disagree	Neither agree nor disagree	Agree	Strongly agree
33.1	International organizations (for example World Health Organization)	1	2	3	4	5
33.2	Ministry of Health	1	2	3	4	5
33.3	Institute for Public Health of Serbia	1	2	3	4	5
33.4	Management/superiors in the health care institution where you work	1	2	3	4	5
33.5	Other health professional	1	2	3	4	5
33.6	Family member or another close person	1	2	3	4	5

34. How much do you agree or disagree that the following factors are important for your personal decision to get vaccinated (or could be important)?

		Strongly disagree	Disagree	Neither agree nor disagree	Agree	Strongly agree
34.1	Concern/fear from getting influenza	1	2	3	4	5
34.2	Concern/fear that my patients and/or colleagues could get infected with influenza if I get sick from influenza	1	2	3	4	5
34.3	Concern/fear that my family members get influenza	1	2	3	4	5
34.4	Concern/fear from being absent from work (sick-leave)	1	2	3	4	5
34.5	Previously had influenza	1	2	3	4	5
34.6	Accessibility/availability of scientific evidence on influenza vaccination	1	2	3	4	5
34.7	Assurances that the vaccine against influenza is safe (person or institution)	1	2	3	4	5
34.8	Assurances from a person I trust that vaccination against influenza can protect me from getting influenza	1	2	3	4	5
34.9	Possibility to get vaccinated at the working place (at the institution where you work_	1	2	3	4	5
34.10	Recommendation from a medical doctor	1	2	3	4	5
34.11	Possibilities of adverse effects of vaccination	1	2	3	4	5
34.12	Reminders from the management	1	2	3	4	5
34.13	Information on when and where I can get vaccinated against influenza	1	2	3	4	5
34.14	Distance from vaccination site	1	2	3	4	5

35. Below are listed statements on influenza and on vaccination against influenza. After carefully reading each statement please circle the response on how much you agree or disagree with the statement.

		Strongly disagree	Disagree	Neither agree nor disagree	Agree	Strongly agree
35.1	Influenza can cause severe disease or death					
35.2	Compared with the general population I have a higher probability to get infected with influenza as I am a health worker	1	2	3	4	5
35.3	Compared with other professions, health workers more often transmit influenza to members of their household	1	2	3	4	5
35.4	Vaccination of health professionals can reduce absence from work (absenteeism)	1	2	3	4	5
35.5	Vaccination of health workers can reduce spreading of influenza among their patients	1	2	3	4	5
35.6	All health care workers employed in health service in Serbia should get vaccinated against influenza	1	2	3	4	5
35.7	Health workers should promote vaccination against influenza	1	2	3	4	5
35.8	Vaccination against influenza is a professional responsibility of health care workers	1	2	3	4	5
35.9	I have sufficient knowledge to be able to respond to patients' questions related to vaccination against influenza	1	2	3	4	5
35.10	In the next (influenza) season (2024/2025) I will recommend vaccination against influenza to persons under higher risk of complications from influenza	1	2	3	4	5

36. What are the most important reasons why you would ACCEPT to get vaccinated against influenza?

37. What are the most important reasons why you would REFUSE to get vaccinated against influenza?

38. Do you have any comment or suggestions with regard to vaccination against influenza of health workers?

THANK YOU FOR TAKING PART IN THIS SURVEY!
